# Editorial: Oncogenic RAS-Dependent Reprogramming of Cellular Plasticity

**DOI:** 10.3389/fonc.2020.00588

**Published:** 2020-04-22

**Authors:** Georgia Konstantinidou, Alessandro Rimessi

**Affiliations:** ^1^Institute of Pharmacology, University of Bern, Bern, Switzerland; ^2^Section of Pathology, Oncology and Experimental Biology, Laboratory for Technologies of Advanced Therapies, Department of Medical Sciences, University of Ferrara, Ferrara, Italy

**Keywords:** oncogenic Ras, cellular plasticity, cancer hallmarks, therapy resistance, oncogenesis

In human cells, three RAS genes, named HRAS, KRAS, and NRAS, encode four highly homologous small GTPases (H-RAS, K-RAS4A, K-RAS4B, and N-RAS). Gain-of-function mutations occur in ~30% of all human cancers, including non-small cell lung cancer, pancreatic, colorectal and breast cancer, and are associated with poor clinical prognosis and resistance to treatment. Since 1982, when activated and transforming human *RAS* genes were discovered, there have been many unsuccessful attempts to target RAS oncogenes. RAS oncogenes have thus long been considered to be undruggable.

Approaches to target RAS oncogenes and RAS-driven cancers are underway, all the efforts to design therapeutics that selectively target the oncogene or its downstream effectors are justified by the degree to which RAS-driven tumors remain dependent on oncogenic RAS, making it a crucial target (1). At the clinical level, the complexity and the signaling redundancy of RAS function and of its downstream pathways have restrained the successful targeting of RAS-mediated oncogene addiction. Although recent discoveries have generated interest in the development of KRAS inhibitors either targeting directly mutant KRAS or targeting the crucial steps required for KRAS activation, these developments can be beneficial only to a small subset of human tumors (2, 3).

RAS proteins principally localize in close proximity to plasma membrane, which participate to the transduction of extracellular growth factor-dependent signaling triggering the activation of different intracellular pathways, such as MAPK and PI3K pathways (4). The lack of functional redundancy between the 3 different RAS isoforms is due to their distinctive intracellular localization and redistribution, generating specific compartmentalized signals (5, 6). Oncogenic RAS signaling establishes cancer hallmark traits that support cancer plasticity, evade immune attack and enhance cancer cell migration and metastasis (7, 8). Moreover, RAS proteins promote metabolic reprogramming of tumor cells, shifting them toward an anabolic metabolism necessary to produce biomass to support their needs (9–12). The specific rewiring depends on the subcellular, cellular, and tissue environments within which oncogenic RAS operates (13).

This Research Topic entitled “*Oncogenic RAS-dependent reprogramming of cellular plasticity*” aimed to contribute to a better understanding of oncogenic RAS signaling in several traits of cancer hallmarks, which are the basis of the reprogramming of cancer cells. The published original research and review articles are briefly described below:

- Muñoz-Maldonado et al. focused on the differences of individual RAS-mutated variants related to signaling and phenotype, as well as on transcriptomics, proteomics, and metabolomics profiles and discussed the association of these mutations with particular therapeutic patient outcomes.
- Galiè reviewed the studies that explored the controversial role of Ras proteins and their mutational status in breast cancer, revealing their role as supporting actors.
-Gimple and Wang reviewed the role of oncogenic RAS and its downstream effectors in different cancer types and grades, focusing on the new strategy of targeting RAS recently emerged and their therapeutic potential.
- Arner et al. reviewed the role of KRAS signaling in epithelial-to-mesenchymal transition (EMT) and cellular plasticity, and discussed the contribution of cellular plasticity in cancer progression, metastasis, and therapy resistance.
- Yang et al. reviewed the recent advances in KRAS-mutant lung cancer with a particular focus on mechanistic insights into tumor heterogeneity, clinic implications, and new therapies.
- Roncarati et al. reviewed the role of microRNAs in RAS oncogenic activation in human cancers, resulting to a potentially useful approach to control RAS oncogenic activation.
- Maffeis et al. reviewed the role of RAS in colorectal cancer and its link with cellular plasticity, invasion, and migration at both molecular and morphological levels.
-Nussinov et al. reviewed the mechanisms through which oncogenic RAS activates its effectors MAPK (Raf/MEK/ERK) and PI3K (PI3K/Akt/mTOR), shedding light on the implications for their pharmacological targeting.
- Pupo et al. reviewed the interplay between KRAS and metabolism focusing on metabolic dependencies of mutant KRAS-driven lung and pancreatic cancers that could be attractive therapeutic targets.

There has been a tremendous progress in the understanding of the genetic architecture, the biological heterogeneity, and the distinct molecular pathways driven by RAS oncogenes that raised new hopes for personalized cancer treatment. More extensive understanding of the RAS pathway in human cancer will guide the future development of precision therapies.

## Author Contributions

GK and AR conceived the idea and wrote the manuscript.

## Conflict of Interest

The authors declare that the research was conducted in the absence of any commercial or financial relationships that could be construed as a potential conflict of interest.

## References

[1] Torti D Trusolino L. Oncogene addiction as a foundational rationale for targeted anti-cancer therapy: promises and perils. EMBO Mol Med. (2011) 3:623–36. 10.1002/emmm.201100176 21953712 PMC3377106

[2] Canon J Rex K Saiki AY Mohr C Cooke K Bagal D. The clinical KRAS(G12C) inhibitor AMG 510 drives anti-tumour immunity. Nature. (2019) 575:217–23. 10.1038/s41586-019-1694-1 31666701

[3] Hallin J Engstrom LD Hargis L Calinisan A Aranda R Briere DM The KRAS(G12C) inhibitor MRTX849 provides insight toward therapeutic susceptibility of KRAS-mutant cancers in mouse models and patients. Cancer Discov. (2020) 10:54–71. 10.1158/2159-8290.CD-19-1167 31658955 PMC6954325

[4] Downward J. Targeting RAS signalling pathways in cancer therapy. Nat Rev Cancer. (2003) 3:11–22. 10.1038/nrc969 12509763

[5] Omerovic J Prior IA. Compartmentalized signalling: Ras proteins and signalling nanoclusters. FEBS J. (2009) 276:1817–25. 10.1111/j.1742-4658.2009.06928.x 19243428

[6] Rimessi A Marchi S Patergnani S Pinton P. H-Ras-driven tumoral maintenance is sustained through caveolin-1-dependent alterations in calcium signaling. Oncogene. (2014) 33:2329–40. 10.1038/onc.2013.192 23728347

[7] Campbell PM Der CJ. Oncogenic Ras and its role in tumor cell invasion and metastasis. Semin Cancer Biol. (2004) 14:105–14. 10.1016/j.semcancer.2003.09.015 15018894

[8] Coelho MA De Carne Trecesson S Rana S Zecchin D Moore C Molina-Arcas M. Oncogenic RAS signaling promotes tumor immunoresistance by stabilizing PD-L1 mRNA. Immunity. (2017) 47:1083–99 e1086. 10.1016/j.immuni.2017.11.016 29246442 PMC5746170

[9] Kamphorst JJ Cross JR Fan J De Stanchina E Mathew R White EP. Hypoxic and Ras-transformed cells support growth by scavenging unsaturated fatty acids from lysophospholipids. Proc Natl Acad Sci USA. (2013) 110:8882–7. 10.1073/pnas.1307237110 23671091 PMC3670379

[10] Padanad MS Konstantinidou G Venkateswaran N Melegari M Rindhe S Mitsche M. Fatty acid oxidation mediated by Acyl-CoA synthetase long chain 3 is required for mutant KRAS lung tumorigenesis. Cell Rep. (2016) 16:1614–28. 10.1016/j.celrep.2016.07.009 27477280 PMC4981512

[11] Rossi Sebastiano M Konstantinidou G. Targeting long chain Acyl-CoA synthetases for cancer therapy. Int J Mol Sci. (2019) 20:3624–39. 10.3390/ijms20153624 31344914 PMC6696099

[12] Saliakoura M Reynoso-Moreno I Pozzato C Rossi Sebastiano M Galie M Gertsch J. The ACSL3-LPIAT1 signaling drives prostaglandin synthesis in non-small cell lung cancer. Oncogene. (2020) 39:2948–60. 10.1038/s41388-020-1196-5 32034305 PMC7118021

[13] Rimessi A Pedriali G Vezzani B Tarocco A Marchi S Wieckowski MR. Interorganellar calcium signaling in the regulation of cell metabolism: a cancer perspective. Semin Cell Dev Biol. (2020) 98:167–80. 10.1016/j.semcdb.2019.05.015 31108186

